# Design of a Protective Single-Dose Intranasal Nanoparticle-Based Vaccine Platform for Respiratory Infectious Diseases

**DOI:** 10.1371/journal.pone.0017642

**Published:** 2011-03-03

**Authors:** Bret D. Ulery, Devender Kumar, Amanda E. Ramer-Tait, Dennis W. Metzger, Michael J. Wannemuehler, Balaji Narasimhan

**Affiliations:** 1 Department of Chemical and Biological Engineering, Iowa State University, Ames, Iowa, United States of America; 2 Center for Immunology and Microbial Disease, Albany Medical College, Albany, New York, United States of America; 3 Department of Veterinary Microbiology and Preventive Medicine, Iowa State University, Ames, Iowa, United States of America; University of California, Merced, United States of America

## Abstract

Despite the successes provided by vaccination, many challenges still exist with respect to controlling new and re-emerging infectious diseases. Innovative vaccine platforms composed of adaptable adjuvants able to appropriately modulate immune responses, induce long-lived immunity in a single dose, and deliver immunogens in a safe and stable manner via multiple routes of administration are needed. This work describes the development of a novel biodegradable polyanhydride nanoparticle-based vaccine platform administered as a single intranasal dose that induced long-lived protective immunity against respiratory disease caused by *Yesinia pestis*, the causative agent of pneumonic plague. Relative to the responses induced by the recombinant protein F1-V alone and MPLA-adjuvanted F1-V, the nanoparticle-based vaccination regimen induced an immune response that was characterized by high titer and high avidity IgG1 anti-F1-V antibody that persisted for at least 23 weeks post-vaccination. After challenge, no *Y. pestis* were recovered from the lungs, livers, or spleens of mice vaccinated with the nanoparticle-based formulation and histopathological appearance of lung, liver, and splenic tissues from these mice post-vaccination was remarkably similar to uninfected control mice.

## Introduction

Natural infections with pathogens stimulate protective and lasting antibody responses because they induce affinity maturation of B cells, a process by which B cells produce antibodies with an increased affinity for antigen during the course of an immune response [1]. Vaccines have been designed to mimic the immune response associated with an active infection yet avoid the undesirable effects of disease. By employing a priming dose followed by two to three booster doses, modern vaccine regimens facilitate the process of affinity maturation, which occurs with repeated or sustained exposure to the same antigen [1]. Vaccines also utilize adjuvants to improve immunogenicity by providing pro-inflammatory signals and prolonging the persistence of vaccine antigens [2]. Unfortunately, current adjuvants approved for human use are not tunable and, as many pathogens have evolved to evade the host immune response, currently available vaccine strategies may not provide adequate induction of long-lived protective immunity. Development of single-dose, tailored nano-adjuvant platforms will not only provide an effective means to induce protective immunity, but will also allow production of cost-effective vaccines that can reduce the need for multiple injections and result in greater patient compliance. Moreover, these novel technologies will obviate the need for hypodermic needles and professionals to administer the vaccine. In this regard, implementation of vaccine delivery systems based on biodegradable polymers offers significant advantages for immunization regimens.

In order to enhance vaccine efficacy and induce long-term, protective immunity, the choice of route (intramuscular [3], [4], subcutaneous [5], [6] or intranasal [4], [7]), adjuvant (Alhydrogel [5], [6], viral vectors [3], polyester microparticles [4], or lipid A mimetics [7]), and vaccination schedule (single-dose [4], [5], [7] or multiple-doses [3], [6]) must all be considered. For respiratory pathogens such as *Yersinia pestis*, intranasal vaccination offers many advantages over parenteral vaccination, including ease of administration and ability to enhance both mucosal and systemic immune responses [8]. While the rapid induction of protection is critical, the ability of vaccine formulations to induce long-lasting protection characterized by high-avidity antibody is equally important [1]. *Y. pestis*, the causative agent of pneumonic plague, is a Category A agent (http://www.bt.cdc.gov/agent/agentlist-category.asp) to which there is no vaccine currently in production. The pursuit of a protective plague vaccine has evolved from the use of killed whole-cell [9] and live-attenuated bacteria [10] to recombinant proteins such as caf1 (i.e., F1) and LcrV (i.e., V) [3], [5], [7]. Previous studies have shown that F1 and V antigen-specific IgG1 facilitates antigen presenting cell phagocytosis and blocks the *Y. pestis* type III secretion system, respectively, leading to protection [11]. In addition, immunization with the fusion protein, F1-V, provides protection in mice [5] and cynomolgus macaques [6]; however, it has been less successful in other non-human primate models such as the African green monkey [12]. To date, only lipid A mimetic adjuvants have been shown to provide long-term, protective immunity against lethal *Y. pestis* challenge [7].

Multiple biodegradable polymers, including polyesters, have been studied as vaccine delivery vehicles [4], [13]. By comparison, the controlled release and adjuvanticity provided by novel polyanhydride carriers, first pioneered by Robert Langer of MIT in the 1980s [14], [15], allows for immune system activation, reduction of antigenic dose, prolonged antigen exposure, stability of the encapsulated protein antigen, and immune modulation [16]–[25]. The results presented herein demonstrate that encapsulation of F1-V into polyanhydride nanoparticles administered as a single intranasal dose successfully induced long-term protection against *Y. pestis* that correlated with a high titer, high avidity F1-V-specific antibody response.

## Results

### Polyanhydride Nanoparticle Design

**Figure 1:**
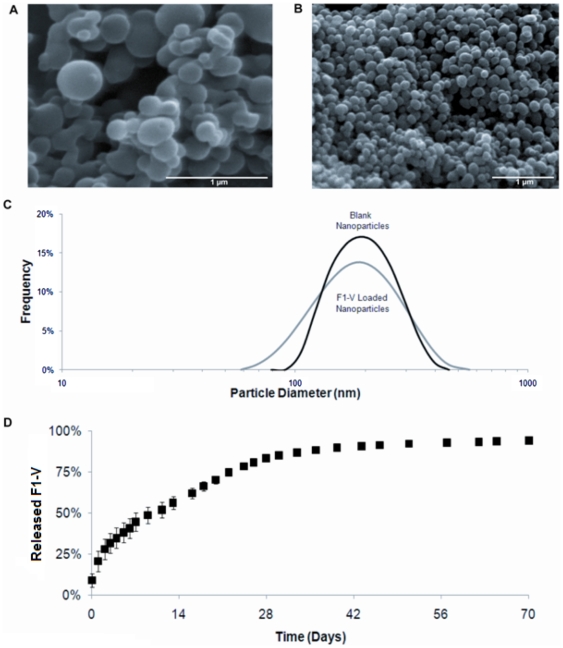
Material properties of 50∶50 CPTEG:CPH nanoparticles. Representative scanning electron photomicrographs of (**A**) blank and (**B**) 2% F1-V loaded 50∶50 CPTEG:CPH nanoparticles (scale bar  =  1 µm). (**C**) Particle size distribution as determined by QELS for blank (204±62) and 2% F1-V loaded 50∶50 CPTEG:CPH nanoparticles (196±77) with n  =  3. (**D**) *In vitro* cumulative release of F1-V from 50∶50 CPTEG:CPH nanoparticles in pH 7.4 PBS analyzed by micro bicinchoninic acid assay (n  =  2, representative of two separate nanoparticle batches).

We have previously shown that encapsulation of F1-V into amphiphilic polyanhydride particles based on 1,6-bis(*p*-carboxyphenoxy)hexane (CPH) and 1,8-bis(*p*-carboxyphenoxy)-3,6-dioxaoctane (CPTEG) successfully preserved the antigenicity of F1-V upon release [26]. Scanning electron photomicrographs of blank and 2% F1-V loaded 50∶50 CPTEG:CPH nanoparticles (Figure 1A and 1B, respectively) show similar spherical morphology and size, which was confirmed by QELS analysis (Figure 1C), demonstrating that antigen encapsulation did not change nanoparticle size (204 nm versus 196 nm). The release kinetics of encapsulated F1-V was monitored for 70 days and was characterized by an initial burst (9%), an approximate zero order release through 28 days, and near complete release (93%) by 70 days (Figure 1D).

### Nanovaccine Protection Against Live Challenge

**Figure 2:**
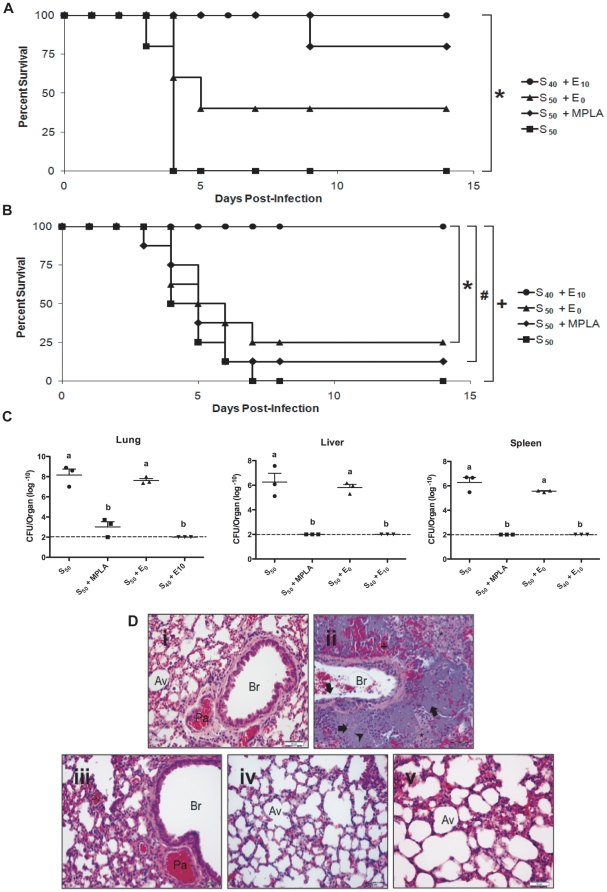
Single-dose, intranasally administered nanovaccines induced protection against lethal *Y. pestis* challenge. C57BL/6 mice were intranasally challenged with 850 CFU (LD_100_) *Y. pestis* CO92 at (**A**) 6 weeks post-vaccination (n = 5 per group) or (**B**) 23 weeks post-vaccination (n = 7 per group) with each challenge a representation of two independent experiments. *  =  p<0.007, #  =  p<0.001 and +  =  p<0.0001. (**C**) CFU of *Y. pestis* CO92 at 72 h post-infection in the lungs, livers, and spleens of mice (n = 3 per group) that were vaccinated 6 weeks prior to challenge. Treatments with different letters are significantly different from one another at p<0.05. (**D**) Photomicrographs of lung sections from mice: uninfected and unvaccinated (i) and challenged 6 (ii, iii, and iv) and 23 (v) weeks post-vaccination. S_50_ vaccinated mice, 72 h post-challenge (ii) showed severe pathology and loss of tissue architecture due to overwhelming bacterial replication in lungs (arrows), neutrophilic infiltration (arrowhead), hemorrhage (+), edema (asterisks), and necrosis. Bronchioles had bacteria clumped with fibrin deposits and neutrophils. Absence of lung pathology was seen in S_40_ + E_10_ vaccinated mice at 72 h (iii), 14 days (iv), and 21 days post-challenge (v). Av - alveolus, Br - bronchiole, Pa - pulmonary artery, Pv - pulmonary vein. Objective lens magnification is 40X. Scale bar  = 50 µm.

To study the effectiveness of antigen-encapsulated nanoparticles to provide protection against pneumonic plague, C57BL/6 mice were intranasally vaccinated (Table 1) and subsequently challenged at 6 weeks or 23 weeks post-vaccination with 850 CFU of *Y. pestis* CO92. At 6 weeks post-vaccination, none of the mice vaccinated with 50 µg of soluble F1-V (S_50_) survived the challenge, whereas 80% of mice treated with S_50_ + MPLA and 40% of mice treated with S_50_ adjuvanted with blank nanoparticles (S_50_ + E_0_) survived (Figure 2A). In contrast, 100% of mice treated with 40 µg of soluble F1-V and 10 µg of encapsulated F1-V (S_40_ + E_10_) survived. At 23 weeks post-vaccination, only 12.5% of mice treated with S_50_ + MPLA and 25% of mice treated with S_50_ + E_0_ survived challenge, in comparison to 100% survival of the mice vaccinated with S_40_ + E_10_ (Figure 2B).

**Table 1:** Vaccination regimens.

Experimental Group	Soluble F1-V (µg)	Encapsulated F1-V (µg)	50∶50 CPTEG:CPH Nanoparticles (µg)	MPLA (µg)
S_50_	50	-----	-----	-----
S_50_ + MPLA	50	-----	-----	10
S_50_ + E_0_	50	-----	500	-----
S_40_ + E_10_	40	10	500	-----

*Quantities indicate the amounts of immunogen or adjuvant delivered to each mouse in the indicated group. S  =  soluble protein; E  =  encapsulated protein. Subscripts indicate amount of soluble or encapsulated protein (in µg) administered per dose.

### Nanovaccine Prevents Pathological Damage Following *Y. pestis* Intranasal Challenge

**Figure 3:**
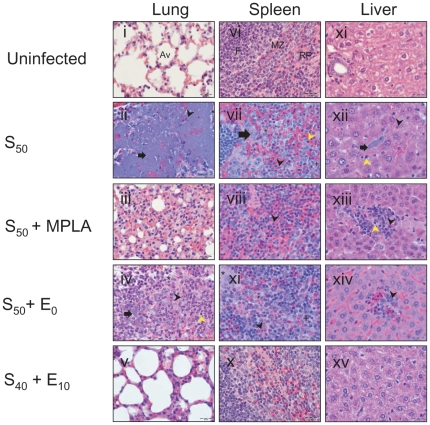
Histopathological analysis of lungs, spleens, and livers 6 weeks post-vaccination at 72 h post-challenge from mice vaccinated six weeks earlier. Lungs of S_40_ + E_10_ vaccinated mice (v) were free of any histopathological lesions and bacteria and were similar to lung tissue from unimmunized and uninfected control mice (i). No bacteria, necrosis or edema were present in spleens of the S_40_ + E_10_ vaccinated mice (x) and histology was similar to healthy spleen tissue (vi). The livers of the S_40_ + E_10_ vaccinated mice (xv) did not show any lesions similar to control mice (xi). Av - Alveoli, F - Follicle, Mz - Marginal zone, and RP - Red pulp. Arrow - bacteria, black arrowhead - neutrophilic infiltration, yellow arrowhead - lymphocytic infiltrations, * – edema, and + - necrotic cells. Objective lens magnification is 100X. Scale bar  = 20 µm.

At 72 h post-infection, bacteriological burdens and histopathological lesions of lungs, livers, and spleens were assessed. The S_50_ + MPLA vaccine regimen prevented bacterial replication in lungs, livers, and spleens at 6 weeks post-vaccination (Figure 2C). However, these mice were not protected at 23 weeks post-vaccination (Figure 2B), suggesting an inability to effectively control bacterial burden. No bacteria were recovered from the lungs, livers, or spleens of mice vaccinated with the S_40_ + E_10_ regimen (Figure 2C). Histopathological appearance of lung, liver, and splenic tissues from mice immunized with S_40_ + E_10_ was remarkably similar to uninfected control mice at both 6 and 23 weeks post-vaccination (Figures 2D and 3). The histopathology data of the liver and splenic tissues in Figure 3 indicates that the single intranasal vaccination not only protected the lungs but also other systemic organs from damage by *Y. pestis*. In contrast, extensive edema and neutrophilic and lymphocytic infiltration were observed in lung, liver, and spleen recovered from mice vaccinated with S_50_, S_50_ + MPLA, or S_50_ + E_0_.

### A Nanovaccine Regimen Composed of Soluble and Encapsulated F1-V Stimulates Enhanced Antibody Production

**Figure 4:**
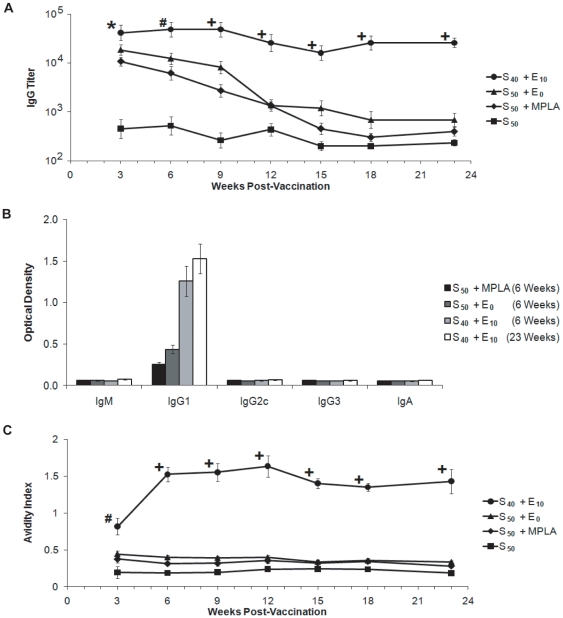
Single-dose administration of nanovaccines induced long-term antibody titers with high avidity. (**A**) Kinetics of IgG antibody titer throughout 23 weeks post-vaccination. (**B**) Antibody isotype induced by various immunization regimens. Optical density was determined by ELISA at a 1∶1000 dilution. (**C**) IgG antibody avidity throughout 23 weeks post-vaccination. Avidity was determined via ELISA at a 1∶200 dilution. Data is presented as the mean ± SEM (n = 7 per group) and is representative of two independent experiments. *  =  p<0.02, **#**  =  p<0.005 and **+**  =  p<0.0001 (compared to S_50_ + MPLA).

Prior work has shown that high antibody titers correlate with protection against live *Y. pestis* challenge [3]–[5], [7]. Consistent with a previous report [7], the soluble protein alone failed to provide protection against a lethal challenge, and mice vaccinated with S_50_ generated low F1-V-specific antibody titers. All adjuvanted vaccines (S_50_ + MPLA, S_50_ + E_0_, and S_40_ + E_10_) induced enhanced anti-F1-V titers in comparison to 50 µg of F1-V alone (Figure 4A). By 6 weeks post-vaccination, anti-F1-V IgG titers began to wane in mice vaccinated with S_50_ + MPLA or S_50_ + E_0_. In contrast, the F1-V-specific IgG titer in S_40_ + E_10_ vaccinated mice was maintained for the duration of the study. Analysis demonstrated that IgG1 was the dominant serum antibody isotype produced (Figure 4B). These studies indicate that anti-F1 and anti-V responses are protective and are consistent with previous work [11].

Protection induced by most vaccines has been shown to be primarily antibody-dependent in nature [1]. In addition to antibody titer, the quality of the antigen-specific antibody, including avidity, also determines vaccine efficacy [1]. For example, poor antibody avidity correlated to a lack of protection against a lethal challenge with the bacterial pathogen *Streptococcus pneumoniae* [27]. In the present study, mice immunized with S_50_, S_50_ + MPLA, or S_50_ + E_0_ formulations developed a low avidity F1-V-specific IgG response (Figure 4C). In contrast, mice vaccinated with S_40_ + E_10_ generated a higher avidity anti-F1-V specific IgG antibody by 3-weeks post-vaccination that was maintained throughout the 23-week experiment (Figure 4C).

These results demonstrate that single-dose intranasal administration of a vaccine formulation consisting of soluble F1-V together with F1-V encapsulated into polyanhydride nanoparticles (S_40_ + E_10_) was able to induce high antibody titers with high avidity that correlated to long-term protection against a lethal *Y. pestis* challenge. When compared to vaccination with soluble F1-V administered with blank nanoparticles (S_50_ + E_0_), it is clear that the combination of encapsulated and soluble antigen is critical to the induction and maintenance of long-term, high avidity IgG1 antibody and protection against lethal challenge. With regards to long-term antigen presentation, we have preliminary evidence that demonstrated that 50∶50 CPTEG:CPH nanoparticles persisted in the lungs of mice for at least 28 days after intranasal administration (data not shown). This evidence is also consistent with previous research that has demonstrated that particles in the same size range as used in the current study were predicted to deposit deep within the lung making them ideal for intranasal vaccination [28]. Nanovaccine formulations containing both soluble and encapsulated antigen facilitated the initiation of a primary immune response [29] and sustained antigen delivery that resulted in the induction of long-lived, high antibody titers with affinity maturation of B cells to generate high avidity antibody [30].

## Discussion

The success of modern vaccines has depended largely upon the inclusion of an adjuvant to promote immunogenic responses by activating antigen presenting cells (APCs) and by protecting the antigen from rapid degradation, thereby creating a depot effect for extended immune stimulation [2]. While the *in vivo* mechanisms that govern nanoparticle-mediated enhancement of immune responses have yet to be elucidated, recent research has demonstrated that activation of both complement and APCs plays a key role in how some nanoparticles augment an immune response [31]. Previous work from our laboratories has shown that polyanhydride nanoparticles of various formulations activate dendritic cells (DCs) *in vitro* [18]. It is likely that the adjuvant properties of the polyanhydride nanoparticles activate DCs *in vivo*. These activated DCs would then traffic to the draining lymph node where they facilitate the induction of an adaptive immune response [29]. Clonal expansion of antigen-specific lymphocytes would involve the interaction with injection-site DCs and (soluble) antigen presented by lymph node resident DCs [29]. The importance of adequate soluble antigen during the initiation of the primary immune response and the subsequent processing and presentation by resident DCs may explain why vaccine regimens employing 100% F1-V encapsulated nanoparticles with no soluble F1-V failed to elicit antibody titers over background levels (data not shown). Additionally, the adjuvant activity observed with the use of nanoparticles agrees with our previous research showing the capacity for nanoparticles to increase soluble protein uptake by APCs *in vitro* [20]. We have also shown that serum proteins adsorb to polyanhydride particles, so it is likely that some F1-V protein does adsorb to the nanoparticles [32]. However, it is currently unknown whether antigen adsorption to the nanoparticle or nanoparticle interactions with antigen presenting cells induces more efficient uptake, processing, and presentation of soluble antigen.

Previous work has shown that single-dose, subcutaneous immunization against *Y. pestis* is not sufficient to induce protection against pneumonic plague [33] while adjuvanted vaccines administered in multiple doses induce high avidity antibody associated with affinity maturation of B cells [34]–[36]. The ability to persist within the body coupled with the delayed degradation of F1-V loaded polyanhydride nanoparticles likely enhanced mechanisms that facilitated extended antigen release and induction of high titer and high avidity antibody responses associated with antigen-specific affinity maturation of B cell responses. While nanoparticles are readily internalized by APCs *in vitro* [18], [20], some particles are not taken up by professional phagocytes. Delayed APC internalization, gradual polymer degradation, and release of antigen would allow for extended presence of antigen. With this in mind, we vaccinated mice intranasally with 200 nm nanoparticles, thus enhancing the potential for these particles to remain in the lung tissue and provide extended antigen delivery [26]. The potential for extended antigen delivery by 50∶50 CPTEG:CPH nanoparticles can be attributed to their presence in the lungs 28 days post-intranasal vaccination as observed using *in situ* fluorescence imaging (data not shown).

To our knowledge, the work presented herein is the first report of a single-dose, synthetic particle-based vaccine to provide long-term protection against lethal challenge of an infectious agent. Moreover, this protective response was characterized by a high titer, high avidity antibody response. Other work from our laboratories has shown that mice vaccinated intramuscularly with a single dose comprised of both soluble tetanus toxoid (TT) and TT encapsulated in polyanhydride microparticles also demonstrated a high titer, high avidity antibody, providing another example of polyanhydride particle-based vaccines enhancing the quality of antibody responses that maximize vaccine efficacy [18]. We propose that the nanoparticle technology described herein can function as an effective delivery platform for a wide range of antigens due to the versatility of the polyanhydride chemistries to stabilize encapsulated proteins, activate APCs, and provide extended release of antigens. Moreover, the scalability of the particle fabrication process enables the design of combination vaccines with customized cocktails of microparticles and nanoparticles of tailored chemistries to be delivered in a single administration. Together, these attributes make polyanhydride particles an attractive platform for development of highly efficacious vaccines.

## Materials and Methods

### Polymer Synthesis and Characterization

Synthesis of 1,6-bis(*p*-carboxyphenoxy)hexane (CPH) and 1,8-bis(*p*-carboxyphenoxy)-3,6-dioxaoctane (CPTEG) diacids was performed as described previously [37]. Gel permeation chromatography and differential scanning calorimetry were utilized to measure molecular weight and glass transition temperature, respectively. The 50∶50 CPTEG:CPH copolymer had a M_n_ of 8,500 Da, PDI of 1.70, and a T_g_ of 13°C, consistent with previous work [37].

### Nanoparticle Design

Both F1-V encapsulated and blank nanoparticles were fabricated by the polyanhydride anti-solvent nanoencapsulation (PAN) method modified from the protocol reported in Ulery et al [21]. For encapsulated nanoparticles, recombinant F1-V (NIH Biodefense and Emerging Infections Research Resources Repository, Manassas, VA) was used. This procedure yielded a fine powder with at least 70% recovery and protein encapsulation efficiency greater than 94%. Nanoparticle morphology was investigated using scanning electron microscopy (SEM, JEOL 840A, JEOL Ltd., Tokyo, Japan). Quasi-elastic light scattering (QELS) was employed to determine nanoparticle size (Zetasizer Nano, Malvern Instruments Ltd., Worchester, UK).

To measure F1-V release kinetics *in vitro*, nanoparticles (12.5 mg) were suspended in 0.1 M phosphate buffer (pH 7.4) at 37°C and agitated. Sodium azide was added to prevent microbial contamination. Aliquots of supernatant were collected at indicated time points and replaced with fresh buffer. Supernatant concentrations were quantified via micro bicinchoninic acid (micro BCA) assay. Total protein encapsulated was determined by adding the quantity of protein released during the experiment to the quantity of protein extracted from remaining nanoparticles as described previously [20]. Cumulative release profiles were generated by normalizing the data against the total amount of encapsulated protein and reported as fractional protein release.

### Bacteria

*Y. pestis* CO92 (NR-641, Biodefense and Emerging Infections Research Resources Repository, NIAID, NIH) was grown overnight at 37°C in heart-infusion broth supplemented with 0.2% D-galactose.

### Mice

Eight-week-old female C57BL/6 mice were obtained from the Jackson Laboratory (Bar Harbor, ME) and maintained under SPF conditions. The Institutional Committees on Animal Care and Use at either Iowa State University or Albany Medical Center approved all procedures involving animals.

### Vaccinations and Challenge

Prior to vaccination, mice were deeply anesthetized via intraperitoneal injection of a ketamine/xylazine cocktail. Mice were vaccinated intranasally with regimens described in Table 1 in a volume of 40 µL. Whole blood was collected from mice via the saphenous vein at the indicated times post-vaccination and serum was assessed for anti-F1-V specific antibodies. For challenge studies, vaccinated mice were anesthetized and challenged intranasally with 850 CFU (LD_100_) of *Y. pestis* in 20 µL PBS/mouse at 6 or 23 weeks post-vaccination. These experiments were conducted in duplicate with a different batch of nanoparticles used for each experiment.

### Quantification of Bacterial Burden and Histopathology

To assess bacterial organ burdens at 72 h post-infection, lungs, livers, and spleens were collected at 6 weeks post-vaccination, homogenized in PBS, diluted ten-fold, and plated on to Congo red agar plates. *Y. pestis* colonies were enumerated after 48 h of incubation at 28°C. Bacterial burdens were expressed as log_10_ means of CFU ± standard errors of the means for three mice per group. Bacterial organ burdens were assessed at 14 days post-infection at both 6- and 23-weeks post-vaccination.

For histopathological studies, vaccinated and infected mice were sacrificed using pentobarbital sodium and lungs, livers, and spleens were collected at indicated time points post-infection and fixed in 10% buffered formalin. Tissues were paraffin-embedded, sectioned, and stained with hematoxylin and eosin. Photomicrographs of tissue sections were acquired and analyzed using cellSens™ standard software (version 1.3, Olympus Corporation, Japan) on an Olympus BX-41 light microscope equipped with Olympus microscope digital camera DP72. Tissues were analyzed histopathologically for evidence of inflammation, hemorrhage, edema, necrosis, changes in tissue architecture, and bacteria.

### F1-V Specific Enzyme-Linked Immunosorbent Assay (ELISA)

Microtiter plates were coated overnight with 0.5 µg/mL F1-V, blocked for 2 h at room temperature with 2.5% skim milk in PBS containing 0.05% Tween 20 (PBS-T), and then washed with PBS-T. Sera samples were diluted 1∶200, then serially diluted three-fold in PBS-T with 1% goat serum, and incubated overnight at 4°C. Plates were washed before adding alkaline phosphatase-conjugated goat anti-mouse IgG(H&L) for 2 h. Plates were washed and developed with phosphatase substrate (Sigma 104, Sigma-Aldrich, St. Louis, MO) in 50 mM sodium carbonate and 2 mM magnesium chloride buffer (pH 9.3) for 30 min. Optical density (OD) of each well was measured at 405 nm. Endpoint titers were defined as the greatest dilution where OD was at least twice that of the average OD of normal mouse serum (1∶1,800).

Antibody avidity analysis was performed as described previously [18]. Serum samples (1∶200) were added to microtiter plates coated with F1-V as described above. Avidity index was defined as the concentration of sodium thiocyanate necessary to reduce the OD by 50% compared to wells treated with 0.1 M sodium phosphate.

### Statistical Analysis

Comparisons between treatment groups were made using two-tailed t-tests. Survival data were analyzed using a Log rank Mantel-Cox test. All statistical analyses were performed with GraphPad Prism 5 software (San Diego, CA).
